# Sport Engagement by Accelerometry under Field Conditions in German Adolescents: Results from GINIPlus

**DOI:** 10.1371/journal.pone.0135630

**Published:** 2015-08-20

**Authors:** Maia Smith, Dietrich Berdel, Dennis Nowak, Joachim Heinrich, Holger Schulz

**Affiliations:** 1 Institute of Epidemiology I, Helmholtz Zentrum München–German Research Center for Environmental Health, Neuherberg, Munich, Germany; 2 Department of Pediatrics, Marien-Hospital Wesel, Wesel, Germany; 3 Comprehensive Pneumology Center Munich (CPC-M), German Center for Lung Research, Munich, Germany; 4 Institute and Outpatient Clinic for Occupational, Social and Environmental Medicine, Ludwig-Maximilians-University, Munich, Germany; University of Rome Foro Italico, ITALY

## Abstract

**Introduction:**

Sporting activities differ in their ability to promote moderate-to-vigorous physical activity (MVPA). To assess adolescents’ engagement in sport under field conditions we used accelerometers to measure their MVPA levels during sport. We pay special attention to differences between team and individual sport and between common sports.

**Methods:**

Diary data and 7-day accelerometry from 1054 Germans ages 15–17 were combined to measure physical activity. 1373 diaried episodes of more than 40 common sports were identified from 626 participants and grouped into team and individual sport. We modeled the effect of team and individual sport, and described levels of MVPA and episodes of no MVPA for all recorded sports.

**Results:**

German boys and girls averaged 43 (SD 21) and 37 (SD 24) minutes MVPA per day. Boys got 2.2 times as much MVPA per minute during team compared to individual sport (p<0.0001) but there was no significant difference for girls. Percent of time spent in MVPA during sport ranged from 6% for weight training to 74% for jogging, with individual sports averaging 10–30% and team sports 30–50%. 11% of sport episodes had no MVPA: half of episodes of cycling, 5% of jogging, and none for tennis or badminton. An episode of individual sport was 17 times more likely to have no MVPA than an episode of team sport (p<0.0001).

**Conclusion:**

Under field condition, adolescents were active for only a fraction of diaried sporting time. As measured by accelerometry, individual sport often produced no MVPA. Characteristics of the sport, such as team vs. individual, were more predictive of MVPA than were characteristics of the participant, such as background activity levels.

## Introduction

Physical activity (PA) is known to reduce morbidity and mortality from noncommunicable disease; however, it is generally accepted that many youth are not sufficiently active [1–3]. Since activity habits tend to persist into adulthood [4] and activity declines sharply at adolescence [5], late childhood is a critical period for assessment and targeted intervention [6].

Activity in children and adolescents is generally summarized in minutes of moderate-to-vigorous physical activity (MVPA) per day or week. A typical goal is 60 minutes of MVPA per day [3, 7, 8]. Estimates of compliance levels vary between studies [3, 5] but most studies[9] agree that further increases would be beneficial. Leisure sport is a common intervention to increase MVPA, but the amount of MVPA associated with various sports under field conditions is not completely understood.

Estimates of physical activity levels for different activities are available, which typically are based on metabolic rate assessed by double-labelled water and/or indirect calorimetry [10] and are generally understood as the gold standard of activity assessment. However, laboratory measurements are not directly applicable to field conditions. One obvious difference is that not all dedicated sporting time is spent active: depending on the environment, a large fraction of sporting time may be spent during instruction, waiting one’s turn or otherwise not engaged in the activity. Estimates for the fraction of young people’s leisure sport time spent active range from 13% [11] to under 50% [12] up to 77% [13]. School sport has been better studied than leisure sport, [12] with estimates typically from 20–40% of class time in MVPA, compared with 15–26% for administrative and management tasks. [14] Simply multiplying laboratory-estimated metabolic equivalents by diaried sport time is thus likely to overestimate the amount of MVPA sport participants receive, and likely contributes to the discrepancy [3] between self-reported and objectively measured activity. This large and flexible discrepancy is likely to impede research into the correlates of activity and distort its apparent effects.

In this study we combine diary data on type of sport with accelerometric measurements of physical activity, in order to calculate how much time participants spend in MVPA for a given period of sport. This fraction is a common measure in studies of sport engagement [12, 15], both within and between groups[1, 16]. Thus, our data allow us to compare the percentage of time adolescents are in MVPA for both team and individual sports under field conditions, and to assess differences in engagement between sports.

Our specific goals in this study are:

- To characterize adolescents with and without leisure sport, evaluate the strength of self-selection in determining sport participation, and compare activity on days without sport in order to specifically test the hypothesis of compensation;
- To evaluate predictors of the fraction of leisure-sport time spent in MVPA, with special attention paid to team vs. individual sport and the distinction between predictors of high MVPA and no MVPA;
- To assess the engagement of adolescents in sport under field conditions by objectively measuring the fraction of time in MVPA for various sports.

## Methods

### Study population

Measurements of physical activity (PA) by accelerometry were embedded in the 15-year follow-up of the “German Infant Nutrition Intervention Programme PLUS environmental and genetic influences on allergy development” (GINIPlus). GINIPlus is an epidemiological study, not a clinical trial, and as a result it is not registered at the ISRCTN or ClinicalTrials.gov. Participants were drawn from both nutrition-interventional and observational arms. The GINIplus study was approved by the local Ethics Committees, the Bavarian General Medical Council (Bayerische Landesärztekammer, Munich, Germany) for the study place Munich and the Medical Council of North-Rhine-Westphalia (Ärztekammer Nordrhein, Düsseldorf, Germany) for Wesel. The approval of the Ethics Committees includes the written consent procedure. Written informed consent was obtained from the parents or the legal guardian of all participants. The cohort and recruitment of the participants has been described in detail at the study website and elsewhere, including in PLOS One [16–18]. Briefly, of 5991 newborns enrolled, 3198 participated in the 15-year followup (53%). Of these, 1890 (59%) consented to accelerometry, of whom 1247 (66%) completed successfully and returned the device. Of these, 1054 (85%) provided data of acceptable quality for inclusion, and 626 participated in sport that could be assigned as “team” or “individual” (for definitions see below).

### Accelerometry

Accelerometers (ActiGraph GT3X, Pensacola, Florida) were worn on the dominant hip for 7 days. Activity on measurement days was recorded in an activity diary using a detailed schedule, including time of going to bed, getting up from bed, time and reason of removing the monitor, and leisure-time sport activities, including type of sport. One episode of sport was defined as the time between starting and finishing an episode of dedicated sporting time. It was possible, though uncommon (6.3%), for participants to have two sporting episodes in a single day. The diary does not distinguish between warmups, practice and games. We find it plausible that among participants who passed our stringent data-quality checks, errors in recording time of starting and finishing sport are random and do not correlate with the type of sport.

At least 10 hours of activity recording (weekday) or 7 (weekend) were required for a valid day: at least 3 weekdays and one weekend day were required for valid data. Levels of PA were assigned according to Freedson’s [19] uniaxial cutoffs into four categories—sedentary, lifestyle, moderate, and vigorous activity—on a minute-by-minute basis during waking hours. MVPA is the sum of moderate and vigorous activity. For detailed description of accelerometer protocol, quality control, and data cleaning, see Supporting Information (S1 File) and [16, 20].

Engagement was defined as the percentage of diaried sporting time where the accelerometer indicated that the child was in MVPA, which has been well studied for school physical education, [12] but not as well for leisure sport [11, 13] and hardly at all as a function of participant-level characteristics such as gender, age, body mass index and location (study center; urban Munich or rural Wesel). Particular attention was paid to activity on non-sporting days as an indicator of baseline activity level. Initial analyses were stratified by gender and by team status of the sporting episode, and then combined into a single model with interaction terms as needed.

### Team Sport

In line with the literature, [21] *team* was defined as all sports where participants directly interact with each other, including interaction with opponents as well as teammates. All sports observed and their status as team or individual are listed in Results.

Preliminary analyses found that leisure-sport participants often received no MVPA at all during an episode of sport, requiring this “zero MVPA” to be accounted for statistically. Further analyses found that episodes with zero MVPA appeared to be a phenomenon distinct from those with low MVPA, and that team and individual sport differed significantly in their rate of zero MVPA.

### Statistical analysis

Values were provided as mean plus/minus standard deviation unless otherwise stated; for skewed variables 5^th^ and 95^th^ percentiles were also provided. Comparisons between individual participants (Table 1) were based on Wilcoxon’s two-tailed rank-sum test; comparisons between sport episodes (Tables 2 and 3) used repeated-measures regression to account for within-child correlation. For all analyses statistical significance was assumed at p<0.05. The fraction of sporting time spent in MVPA was modeled both as a binary “any MVPA versus none” and as a rate (minutes in MVPA / total diaried sporting time, when the episode had any MVPA). This separation was statistically necessary and also turned out to be clinically relevant.

**Table 1:** Participants by Sport Participation (Mean, SD).

Predictor	All participants (N = 1054)^1^		Participants with diaried sport (N = 626)		P for difference between those with and without diaried sport^2^	
	Boys	Girls	Boys	Girls	Boys	Girls
Male (N, %)	475 (45)		263 (42)		0.016	
Age (years)	15.7 (0.55)	15.7 (0.54)	15.7 (0.55)	15.7 (0.55)	0.14	0.37
Parents highly educated (%)^3^	64	66	68	69	0.052	0.17
From Munich (urban)	57	50	58	46	0.64	0.027
BMI (kg/m^2^)	20.7 (3.2)	21.0 (2.9)	20.5 (3.0)	21.1 (2.9)	0.40	0.50
BMI category (%)^4^						
Underweight	9.74	6.25	9.88	4.70	0.98	0.048
Normal	79.0	85.2	80.2	85.9	0.45	0.84
Overweight	8.66	5.21	7.91	6.35	0.48	0.10
Obese	2.60	3.30	1.98	3.04	0.33	0.66
Overweight + obese	11.0	8.51	9.88	9.39	0.26	0.31
Activity on all days (min):						
Sedentary^5^	586 (81)	598 (69)	584 (75)	593 (63)	0.39	0.0016
Lifestyle^5^	259 (60)	247 (51)	261 (56)	253 (50)	0.29	<0.0001
Moderate^5^	29.6 (13)	26.0 (15)	32.1 (13)	26.6 (14)	<0.0001	0.00077
Vigorous^5^	13.0 (11)	10.8 (11)	15.2 (11)	11.5 (9.3)	<0.0001	<0.0001
MVPA^5^	42.6 (21)	36.8 (23)	47.3 (20)	38.2 (20)	<0.0001	<0.0001
Total vertical counts, thousands	327 (117)	296 (131)	352 (108)	309 (111)	<0.0001	<0.0001
% days with > 60 minutes MVPA	25 (23)	17 (22)	31 (23)	19 (21)	<0.0001	<0.0001
Activity on non-sport day ^6^ (min)^5^						
Sedentary^5^ ^,^ ^6^	596 (87)	610 (77)	601 (87)	610 (76)	0.13	0.73
Lifestyle^5^ ^,^ ^6^	251 (63)	238 (55)	248 (60)	240 (57)	0.26	0.24
Moderate^5^ ^,^ ^6^	25.9 (13)	23.3 (15)	25.9 (13)	22.5 (13)	0.87	0.33
Vigorous^5^ ^,^ ^6^	9.94 (9.9)	7.85 (11)	10.1 (9.6)	6.90 (7.2)	0.50	0.18
MVPA^5^ ^,^ ^6^	35.8 (20)	31.1 (23)	36.0 (20)	29.4 (18)	0.59	0.23
Total vertical counts, thousands	288 (111)	257 (128)	287 (103)	250 (98.2)	0.83	0.89

^1)^ Cohort previously described in[20]; subset described in [23]

^2)^ Wilcoxon rank-sum test, two-sided

^3)^ Based on maximum of mother and father; 1 if university entrance or higher, 0 otherwise.

^4)^ Based on sex- and age-specific percentiles in[22]; expected percentages would be 10% underweight, 7% overweight, and 3% obese.

^5)^ Activity levels as described in [24]. MVPA is moderate or vigorous.

^6)^ 9 participants (1.4%) had sport every day and are thus excluded from these summary statistics

**Table 2:** Team and Individual Sport Episodes (N = 1373 episodes, 626 participants). 213 participants had only team sport; 332 had only individual sport; 81 had both.

	Team (N = 559 episodes)			Individual (N = 814 episodes)			Differs between team and individual sport episodes? (p)	
	Boys	Girls	P for sex difference	Boys	Girls	P for sex difference	Boys	Girls
**Number of episodes**	331	228	—	239	575	—	—	
**Percent time in MVPA** ^1^ ^**,**^ ^2^			<0.0001			<0.0001	<0.0001	0.37
Mean (SD)	47.8 (22)	33.2 (19)		21.2 (27)	31.3 (29)			
5^th^, 95^th^ percentile	6.2, 80	3.3, 67		0, 83	0, 91			
**Percent time in MVPA when >0** ^1^ ^**,**^ ^2^			<0.0001			<0.0001	<0.0001	0.34
Mean (SD)	48.3 (21)	33.7 (19)		27.5 (27)	36.6 (28)			
5^th^, 95^th^ percentile	6.7, 80	3.4, 67		1.7, 88	2.2, 93			
**Episode had no MVPA** N, (%)^3^	3 (0.91)	3 (1.32)	0.65	55 (23.0)	82 (14.3)	0.0024	<0.0001	<0.0001
**Episode length, minutes** ^1^			0.19			0.26	0.25	0.0071
Mean (SD)	107 (54)	101 (42)		96.4 (105)	85.8 (79)			
5^th^, 95^th^ percentile	55, 195	60, 180		15, 375	20, 225			
**% of sporting time:**								
**Sedentary** ^1^ ^**,**^ ^2^ ^**,**^ ^4^			<0.0001			<0.0001	<0.0001	0.11
Mean (SD)	12.3 (14)	17.8 (15)		28.1 (22)	20.3 (20)			
5^th^, 95^th^ percentile	0, 40	1.7, 49		0, 73	0, 60			
**Lifestyle** ^1^ ^**,**^ ^2^ ^**,**^ ^4^			<0.0001			0.44	<0.0001	0.95
Mean (SD)	39.9 (19)	49.0 (18)		50.7 (24)	48.4 (23)			
5^th^, 95^th^ percentile	13, 77	20, 79		6.7, 87	5.7, 85			
**Moderate** ^1^ ^**,**^ ^2^ ^**,**^ ^4^			<0.0001			0.024	<0.0001	<0.0001
Mean (SD)	27.0 (13)	21.6 (13)		7.79 (9.2)	10.1 (12)			
5^th^, 95^th^ percentile	4.5, 59	2.2, 44		0, 27	0, 33			
**Vigorous** ^1^ ^**,**^ ^2^ ^**,**^ ^4^			<0.0001			0.0038	0.0026	<0.0001
Mean (SD)	20.8 (18)	11.6 (14)		13.4 (25)	21.2 (27)			
5^th^, 95^th^ percentile	0, 51	0, 39		0, 80	0, 88			
**Total counts /minute, thousands**			<0.0001			0.018	0.0023	0.0002
Mean (SD)	24.8 (10)	18.3 (8.4)		17.8 (23)	23.5 (21)			
5^th^, 95^th^ percentile	8.6, 42	5.5, 34		1.5, 81	2.2, 70			

P-values from generalized linear model, with child as repeated measure.

^1)^ Negative-binomial distributed

^2)^ Log total weartime as offset

^3)^ Binary distributed

^4)^ Activity levels as described in [24]. Team sport defined as any requiring interaction with other participants, in line with[21]

**Table 3:** Multivariable Predictors of MVPA in Sport, All Episodes.

Predictor	Predicts fraction of sport time in MVPA, when MVPA>0 N = 575 participants, 1183 episodes		Predicts risk that MVPA = 0 N = 626 participants, 1373 episodes	
	Rate ratio %	P	Odds ratio	P
Intercept (baseline)	20	0.033	0.078	0.40
Age, years	103	0.49	1.11	0.58
Study center Munich	85	0.0028	0.89	0.62
Parental education^1^	107	0.29	0.936	0.79
Male	77	0.0075	1.82	0.0072
Episode length, 10 minutes	--	--	0.89	0.0015
Activity on nonsport days, 10 minutes		--		
**Moderate** ^2^	104	0.0075	--	--
Vigorous^2^	N/A	N/A	--	--
Moderate*Male^2^	N/A	N/A	--	--
Vigorous*Male^2^	N/A	N/A	--	--
**Team sport** ^3^	N/A	N/A	0.0596	<0.0001
**Team** ^3^ *Male	181	<0.0001	N/A	N/A

^1)^ Binary: 1 if better-educated parent entered college, 0 if not.

^2)^ Activity levels as described in [24]. MVPA is moderate or vigorous physical activity and refers to non-sporting days only. Participants with sport every day (9, 1.4%) are missing.

^3)^ Team sport defined as any requiring interaction with other participants, in line with [21]

N/A = P ≥0.05 so predictor was removed from the model.,— = not included in initial model

Predictors above the double line (age, study center, parental education, and gender; episode length when predicting zero MVPA, but not when predicting nonzero MVPA) left in model regardless of statistical significance. Nonzero MVPA modeled with negative binomial regression with log of total sporting time as offset, zeroes with logistic regression, both with child as a repeated measure.

As predictors of engagement we considered the typical factors of age, gender, socioeconomic status (parental education), BMI and BMI category, as well as urban vs. rural location and length of the sporting episode. Many of these are known to correlate with activity levels; location may predict baseline activity, total activity, and/or the specific sporting activities available.

Sociodemographic data, such as parental education and study center, were obtained from questionnaires. As a proxy for socioeconomic status, the maximum of mother’s and father’s education above college entrance when the child was 3 years old (binary yes-no) was modelled as a single predictor: it was kept in the model for consistency with other work with this cohort regardless of statistical significance. Height and weight were generally obtainable from physical examination (n = 964 out of a total of 1054), but when this was missing, self-reports were used. Among participants with both self-reported and objective measures, correlation between the two was very strong: 0.92 for weight and 0.95 for height. Only complete cases were analysed. In addition to its linear effect, BMI was categorized into *underweight*, *normal*, *overweight*, or *obese* according to German reference values [22] for the 10^th^, 90^th^, and 97^th^ percentiles of BMI for age and sex in the reference population.

Because of possible crossover interactions initial analyses were stratified by team status of the episode, and then combined in a single model with interaction terms as necessary.

## Results

### Study population

1054 individuals (45% male) participated in accelerometry, and 626 (42% male) participated in a total of 1373 diaried episodes of leisure sport. Characteristics of the study population and differences between those with diaried leisure sport and the rest of the cohort providing accelerometric data are described in Table 1.

Participants with diaried sport were more active than the cohort average, with higher levels of total vertical counts, moderate, vigorous, and MVPA; differences for MVPA were 10.5% in boys and 3.8% in girls (p<0.0001). However, this difference appeared to be entirely due to the presence of leisure sport itself because on days without diaried sport activity neither moderate nor vigorous activity differed significantly between groups.

Anthropometric and sociodemographic differences between those with and without sport were small and clinically nonsignificant. Furthermore, the data reflect an almost uniformly high standard of living within the cohort, as 2/3 of participants had at least one parent who had entered college. Prevalences of overweight and obesity (expected values 7% and 3%) were typical for 2001, when the reference population was established, in spite of the well-documented rise in adiposity in Germany during this time. Accelerometry completers were also 55% female, more than the rest of the GINI cohort (49%). However, within this subgroup there was little further selection bias for diaried sport. All further analyses were limited to those participants with any diaried leisure sport.

### Team versus Individual Sport Episodes

**Fig 1:**
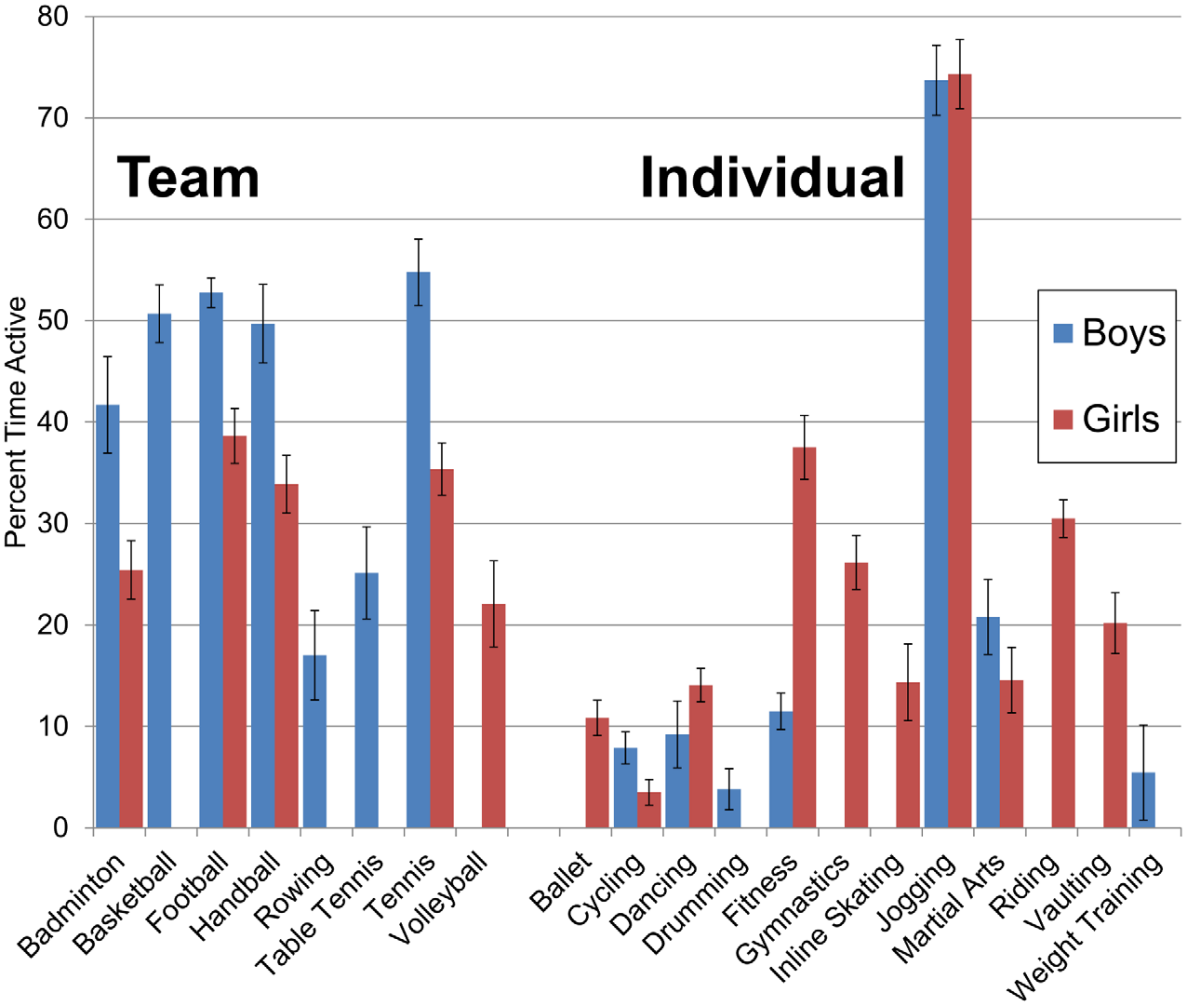
Time in MVPA During Sport. MVPA as determined in Freedson‘s accelerometric algorithm (Freedson et al 2005, #19). Sports shown if that sex had >10 episodes during recording. Error bars for standard error of the mean.

Sporting participants are described in Table 1, while sporting episodes are described in Table 2 and shown in Fig 1. Most participants had only team sport (n = 213) or only individual sport (n = 332): only13% (n = 81) had both. Preliminary analyses found that the only significant difference between groups was gender: boys were more likely to do team sport, while girls were more likely to do individual sport. Therefore, all statistical models and tables are either stratified or corrected for the effect of sex.

On average individual sport had shorter episodes than team, by 15% for girls (p = 0.0066) and 10% for boys (not significant). There was no significant difference in episode length by sex, though girls averaged somewhat shorter episodes than boys in both team and individual sport.

Both sexes got more MVPA in team sport than in individual, though the difference was only significant in boys. Team sport produced 2.2 times as much MVPA per unit time than individual sport in boys (48% of diaried team-sport time was spent in MVPA, vs. 21% of individual, p<0.0001) but there was no significant difference for girls (33 vs 31%). When episodes with no MVPA were excluded, the difference between team and individual sport engagement shrank in boys to 1.76 (p<0.0001) and reversed in girls to 0.92 but remained nonsignificant. Boys in team sport got more MVPA in a given time than girls (48% vs 33%, p<0.0001) but the reverse was true in individual sport where girls were more active (31% vs 21%, p<0.0001). This remained true when episodes with no MVPA were excluded. However, both sexes got more MVPA in team sport than in individual though the difference was only significant in boys.

Individual sport had many more episodes with no MVPA: 55/239 (23%) and 82/575 (14%) of individual-sport episodes had no MVPA for boys and girls, while 3/331 (0.91%) and 3/228 (1.3%) episodes of team sport did. The difference between the rate of episodes with no MVPA in team and individual sport was highly significant (p<0.0001); so is the sex difference in the rate of zero-MVPA episodes within individual sport, though not team sport.

For both sexes, individual sport produced more variable levels of MVPA than team sport. The coefficient of variation was 1.27 for boys in individual sport, compared with 0.46 in team sport, and 0.93 for girls in individual sport compared with 0.57 in team sport. This observation was confirmed when comparing the 5^th^ and 95^th^ percentiles for percentage time in MVPA, which are wider for both sexes in individual sport than in team. These were 3.3% and 67% for girls in team sport, and 0% and 91% in individual sport. When sport episodes with no MVPA were excluded, the percentiles for girls in individual sport were 2.2% and 93%. Comparable results were obtained in boys.

In summary, objective assessment of sports under field conditions showed that team sport generally produced more MVPA per unit time than individual sport in both sexes, clearly in boys but less so in girls. However, the typical effect of gender was reversed in individual sport: girls got more MVPA in individual sport than boys did, and boys had more episodes with no MVPA. Individual sport was more likely than team sport to have episodes with no MVPA, and MVPA levels were more variable in individual sport even when episodes with no MVPA were excluded.

### Multivariable Predictors of Leisure Sport Engagement

Initial models of MVPA during leisure sport episodes were stratified by gender of the child and team status of the episode, but we found that within-child predictors were weak and generally homogeneous between team and individual sport and between sexes. Thus, a combined analysis for multivariable predictors of time spent in MVPA during sport episodes was performed (Table 3) in order to directly estimate the effects of gender and team sport. In the combined model, parameter estimates did not change significantly from the stratified models.

Vigorous and lifestyle activity on non-sporting days did not significantly predict sport engagement in either sex, although moderate activity did: each 10 minutes of daily moderate activity per day was associated with an average 4% increase in MVPA level during sport. (RR 1.04, p = 0.0075). No sociodemographic or anthropometric predictor was significant, except study center (RR = 0.85 for study center Munich, p = 0.0028). Leisure-sport engagement was not significantly predicted by age, BMI whether linear or categorical, parental education level (socioeconomic status), or episode length. MVPA levels in girls did not differ significantly between team and individual, but in individual sport boys had 23% lower MVPA than girls (rate ratio 0.77, p = 0.0075). However, boys in team sport still had significantly more MVPA than girls in team sport because male gender interacts significantly with team sport (rate ratio for male*team interaction 1.81, p<0.0001)

### Predictors of Sport Episodes with No MVPA

An episode of individual sport was 17 times as likely to have no MVPA as one of team sport (Table 3, odds ratio 0.0596 for team sport as compared with individual; p<0.0001). Other risk factors that an episode would have no MVPA were male gender (OR 1.82, p = 0.0072) and short episodes of sport (OR = 0.89 per 10 minutes, p = 0.0015). Sociodemographic predictors (age, parental education and study center) had no effect.

### Estimates of MVPA from Specific Sports

Table 4 presents average fraction of time participants spent in moderate, vigorous and MVPA for all sports in our sample that had 10 or more episodes for at least one sex, including episodes with no MVPA. The remaining sports are in Table 5. “Martial arts” in Table 4 is the combination of judo, karate, aikido and taekwondo. Sex-stratified averages are presented in Fig 1. All values presented here include episodes with zero MVPA and are averaged across episodes of that sport, to better represent total time spent within that sport.

**Table 4:** Engagement in Sports with 10 or More Episodes for at Least One Sex.

	Episodes (number)				Percent time in				
Sport	Zero MVPA	Boys	Girls	Episode length, minutes	Moderate	Vigorous	MVPA	Counts per minute, hundreds	Sex with more MVPA per unit time
**Individual**									
Ballet	0	0	15	76	6	4	10	8.84	--
Cycling	49	43	47	64	4	2	6	6.44	Boys
Dancing	20	17	94	98	8	4	14	9.94	Girls
Drumming	7	13	0	52	4	0	4	2.82	--
Fitness	18	56	88	70	10	16	26	16.9	Girls
Gymnastics	1	4	23	120	16	12	28	17.2	Boys
Inline Skating	9	6	35	78	12	2	12	9.01	Girls
Jogging	5	28	67	36	8	66	74	58.3	Girls
Martial Arts	4	13	15	84	10	6	18	12.9	Boys
Riding	5	1	123	100	8	22	30	27.2	Boys (only one)
Vaulting	0	0	14	118	10	10	20	15.3	--
Weight Training	4	12	5	74	4	2	6	5.80	Boys
**Team**									
Badminton	0	12	26	92	20	10	30	17.8	Boys
Basketball	1	41	10	102	24	26	50	26.7	Boys
Football (soccer)	1	152	53	100	26	22	50	24.9	Boys
Handball	1	26	35	100	24	16	40	21.2	Boys
Rowing	0	15	1	130	10	8	18	14.7	Girls (only one)
Table Tennis	2	19	10	124	18	4	22	14.1	Boys
Tennis	0	44	43	82	32	14	46	22.1	Boys
Volleyball	1	7	23	132	18	6	24	13.9	Boys

Activity levels as described in[24]. MVPA is moderate or vigorous. Moderate + vigorous may not sum to MVPA because of rounding. Team sport defined as any requiring interaction with other participants, in line with [21].

**Table 5:** Engagement in Sports with 9 or Fewer Episodes for Both Sexes.

	Episodes, count				Percent time spent in				
Sport	Zero MVPA (total)	Boys	Girls	Episode length, minutes	Moderate	Vigorous	MVPA	Vertical-axis counts per minute, hundreds	Sex with more MVPA per unit time
**Individual**									
Aikido	0	2	1	74	10	8	16	12.9	Boys
Archery	4	6	2	112	2	0	2	4.08	Boys
Boxing	0	3	0	64	18	30	48	27.9	Boys
Cross-Country Skiing	0	1	0	52	46	6	52	22.4	Boys
Golf	0	2	0	176	24	18	42	21.7	Boys
Hiking	0	0	6	366	52	6	58	20.5	--
Ice Skating	1	3	4	128	8	2	10	9.64	Boys
Judo	1	1	0	2	0	0	0	7.19	Boys
Karate	0	3	4	90	16	6	22	14.1	Boys
Nordic Walking	0	0	1	50	2	88	90	55.6	--
Shooting	5	4	3	44	0	0	2	2.41	Girls
Snowshoeing	0	0	1	180	20	10	30	13.4	--
Swimming	0	0	1	74	4	0	4	3.02	--
Trampoline	1	4	2	54	4	40	46	65.6	Boys
Walking	1	3	9	96	24	8	32	15.3	Boys
Yoga	1	0	2	52	0	0	0	2.4	--
Zumba	0	0	4	52	26	10	36	20.3	--
**Team**									
Baseball	0	1	6	100	14	2	18	13.1	Girls
Fencing	0	0	4	158	6	6	10	8.72	--
Fistball (“Faustball”)	0	3	1	128	14	2	16	10.8	Boys
Ice Hockey	0	4	3	174	6	8	14	14.9	Girls
Rugby	0	0	3	120	14	18	32	17.7	--
Scouting	0	2	0	386	8	2	8	8.50	Boys

Activity levels as described in[24]. MVPA is moderate or vigorous. Moderate + vigorous may not sum to MVPA because of rounding. Team sport defined as any requiring interaction with other participants, in line with [21].

Engagement in individual sports was more variable than in team sports, making up both the largest and smallest fractions of time in MVPA in the sample. During ballet, cycling, drumming, and weight training about 4–10% of time was spent in MVPA while fitness training, gymnastics, and riding produced between 20 and 30. The highest engagement was observed in jogging, with 74% of time in MVPA. For team sport the lowest MVPA values of about 20% were observed during rowing, table tennis, and volleyball, and the highest values, ranging between 45 and 50%, were basketball, football, and tennis. Among common team sports football, basketball, handball, and tennis produced the most MVPA, while jogging, riding, gymnastics and fitness training were the most active individual sports.

We observed an interaction between team sport and sex, with boys being more engaged than girls in team sport but comparable in individual sport when including only episodes with MVPA. Furthermore,boys were less engaged when zero-MVPA episodes of sport (almost twice as prevalent in boys) were included in the average. Fig 1 indicates that this interaction was not driven by any single team or individual sport. The only team sport in which girls averaged more MVPA across all episodes than boys was rowing, and that estimate was based on a single episode for girls and is thus unreliable. The sexes were much more similar for individual sport, with girls more engaged than boys in dancing, fitness, and inline skating and with very similar engagement in weight training and jogging. Thus it appears that while boys were reliably more engaged than girls in most team sports, the sex difference in individual sport as a whole was much weaker and perhaps depends on which sports are included in the sample.

Most individual sports with 10 or more episodes for at least one sex had at least one episode with zero MVPA, even those with otherwise high MVPA such as jogging. The association of individual sport with zero MVPA did not appear to be a result of the specific individual sports in our sample, and it appeared to be distinct from low MVPA. 5/95 episodes of jogging had no MVPA (5%), 49/90 episodes of cycling (54%), 20/111 (18%) episodes of dancing, and 4/28 (14%) episodes of martial arts.

## Discussion

Accelerometric and diary data were combined to assess the engagement in common leisure sports in adolescents under field conditions and determine predictors of MVPA levels for different sports. General physical activity levels of our sample were well within the wide range reported for youth [2, 25–30] but participants with diaried leisure sport had above-average levels of activity. This was entirely due to their participation in sport, since activity on days without leisure sport was comparable between groups. This indicates that outside sporting time, physical activity behavior is similar between adolescents who do and do not participate in sport.

Engagement in leisure sport compared favorably with that in school physical education (school sport) but was still relatively low. Our previous work with this cohort [20] found that 22% of school physical education time was spent in MVPA, which is about two-thirds of what we found for leisure sport. However, engagement in leisure sport was highly variable, with no MVPA at all in many episodes, particularly in individual sport. Engagement in different sports ranged from ^1^/_10_ to ¾ of total time in MVPA. This range is comparable to that reported in the literature for youth. [11] [12][13]. Taken together, these findings imply that much of the difference between accelerometric and self-reported activity levels [3, 10, 31, 32] may be explicable by the assumption that 100% of sport time is spent active.

Our study is comparable to existing literature in recording only sporting time, rather than distinguishing between warmups, practices and games, and our estimates of engagement level can thus be compared with theirs. Furthermore, we are able to compare different sports within the same sample in a way that other studies are not, since they generally either do not record the specific sport or are limited to one, such as Morales et al [13] which estimated that 77% of basketball time was spent in MVPA. While our estimate was only 2/3 that, it was still the highest among team sports. The difference may reflect individual characteristics such as age and gender (our cohort included girls, while Morales et al did not), differences in measurement methods, differential engagement in practice and games, or other factors.

Our observed higher engagement in team sport than individual may result from the well-known Köhler effect, where less-capable members of a group perform better in the group than alone [33] particularly when they are aware that their performance is limiting that of the group. Little research has examined how the effect influences more-capable group members, but we suggest that it may also help explain why girls were less engaged than boys in team sports despite being comparably engaged in individual sports. Since team sport is generally zero-sum, winning may be more motivating for males, who average relatively higher competitiveness [34, 35] and lower agreeability [36] than females. Furthermore, females may consciously lower their engagement as a result of the well-known “likability-competence tradeoff” [37] [38]. Indeed, at least one study [39] found that female, but not male, runners adjusted their performance downward to match a less-capable partner—even when the partner was not only a stranger, but a computer simulation.

It is difficult to make recommendations from this finding, since sport choices are not randomized but are instead based on availability of the sport and personal preference of the participant. However, coaches and participants should be aware of the social interactions generated by peers and coaches during sport, particularly the potentially detrimental effects for females.

More generally, we draw attention to the wide range of engagement levels we observed both within and between sports. To increase both participation and engagement,we suggest increasing the sporting options available and lowering the barriers to participation. We suggest that this will increase the chances that adolescents find sports which they enjoy, find socially acceptable, and are willing to engage in at a high level.

### Study Strengths and Limitations

Our study is favorable in its large sample—626 sport participants and over 1,000 sport episodes, compared with 228 participants for [12] and 14 basketball classes for [13]—in its objective assessment of MVPA, and its documentation of specific sport type. However, study participants were recruited as followup of a birth cohort, and assessed their PA levels through accelerometry and diary, both of which are labor-intensive for the participant. Selection bias is thus likely at all stages, from initial enrollment in the cohort to availability for followup, consent to and completion of accelerometry, and a series of demanding quality checks of the data. Many of our findings, such as the null effects of age and socioeconomic status, almost certainly reflect this; and even within the GINI cohort, earlier work (data in [20]) found associations between accelerometry completion and both healthy weight and female gender. Thus the null finding between SES and physical activity needs a cautious interpretation, because of the potential for bias.

We find no strong effects on PA from any sociodemographic predictor, in spite of the known effects in other populations. However, our goal was not to capture all possible predictors of PA: numerous factors affect PA and many are population-specific and/or difficult to measure objectively. Furthermore, many do not vary much in this relatively high-SES, native-German population and thus their effects could not be estimated within it. We thus checked only those within-participant confounders that are easy to measure and most commonly cited as predictors of activity, and realize that even these effects are specific to our population.

Another limitation is that our estimates of engagement in leisure sport completely rely on the accuracy of diaried sporting time. This limitation is common to all studies of behavior that rely on self-report, and we minimized it as much as possible with a series of stringent quality checks, particularly for inconsistencies between diaried wear time and wear time determined by the NHANES algorithms, to exclude unreliable participants and to minimize inconsistencies. Hence, errors in recording time of starting and finishing sport are likely to be small. Furthermore we did not have any indications that error was associated with sport type, and thus the assumption of random errors is plausible.

This study is fundamentally cross-sectional, measuring variations within a large and homogeneous cohort over a period of about a week per participant. We were unable to estimate longer-term effects, such as seasonal variation or within-participant replicability. However, our study is among the largest of its type (n>1,000 participants) and as far as we know the only one to examine engagement in specific sports under field conditions.

## Conclusions

Objectively-assessed leisure-sport engagement varies substantially between adolescents and between sports under field conditions in Germany. Adolescents are active for only a fraction of diaried sporting time, and episodes of individual sport often appear to have no MVPA. Boys are more engaged than girls in team sport, but are slightly less engaged and much more likely to have no MVPA in an episode of individual sport. Given this gender difference, we suggest that engagement can be maximized by allowing and enabling participants to choose the sporting activities they most enjoy, bearing in mind that team sports may be more preferred by boys and individual sports by girls. We also suggest monitoring and managing social pressures between sporting participants, particularly pressures towards low engagement if these are present.

Adolescents with sport are more active than those without it, but only on days with sport. We found no evidence for activity compensation: MVPA accumulated during sport is simply added onto the daily total.

## Supporting Information

S1 File Technical—Accelerometry and statistics. (DOC)
