# Supplementary material for: Sport Engagement by Accelerometry under Field Conditions in German Adolescents: Results from GINIPlus
Source: PLoS One. 2015 Aug 20;10(8):e0135630. doi: 10.1371/journal.pone.0135630 (PMC4546233; doi:10.1371/journal.pone.0135630)
Supplement: S1 File — (DOC) [file pone.0135630.s001.doc]

*Technical information: Accelerometry and activity diary*

Triaxial accelerometers (ActiGraph GT3X, Pensacola, Florida) were worn on the dominant hip for up to 7 days. Activity on measurement days was recorded in an activity diary using a detailed schedule, including time of going to bed, getting up from bed, time and reason of removing the monitor, and leisure-time sport activities, including type of sport. No standardized timekeeping device was provided and therefore subjects used their own, but it is likely that the activity diary entries for each subject used the same device and therefore errors would tend to cancel out.

At least 10 hours of activity recording (weekday) or 7 (weekend) were required for a valid day: at least 3 weekdays and one weekend day were required for valid data. Ultimately, 1054 subjects (84% of those participating in PA measurements, 45% male) provided usable accelerometric data, of whom 626 had any diaried sport. For detailed description of accelerometer protocol, quality control, and data cleaning, see (Pfitzner 2013) and (Smith 2014).

When the accelerometer was not worn, the time and reason was recorded in the diary. Occasionally this reason was sport with potential to damage the device, which was described as NWT-Sport (nonwear-time due to sport). NWT-Sport was scarce, occurring on less than 5% of recording days. (Data not shown). No attempt was made to impute data for NWT-Sport in the present study, since doing so would likely blur the distinctions between different types of sport. Rather, the total amount of time spent in sport was expressed as total number of minutes between the start and end of sport when the accelerometer was worn. Our estimates of baseline activity may be slightly downward biased as a result, but only slightly.

Levels of PA were assigned according to Freedson’s (Freedson 2005) cutoffs into four categories— sedentary, lifestyle, moderate, and vigorous activity—on a minute-by-minute basis during waking hours. MVPA is the sum of moderate and vigorous activity. Diary data was used to assess activity during sport minute by minute.

626 individuals had diaried sport, with 46 sports and between 1 and 205 episodes of each sport. Statistical analysis was carried out to characterize subjects by presence or absence of sport (Table 1) in order to assess selection bias. Of the 626 individuals with valid sport, 332 had only individual sport and 213 had only team sport. 81 (13%) had both. Of the 1373 diaried episodes of sport, 559 were team and 814 individual.

*Collection and Choice of Confounders*

As a proxy for socioeconomic status, the maximum of mother’s and father’s education above college entrance (binary yes-no) was modelled as a single predictor: it was kept in the model for consistency with other work with this cohort regardless of statistical significance. In addition to its linear effect, BMI was categorized into underweight, normal, overweight, or obese according to German reference values (Kromeyer-Hauschild 2001) for the 10th, 90th, and 97th percentiles of BMI for age and sex in the reference population.

To correct for baseline activity levels, we calculated daily averages of the four levels (sedentary, lifestyle, moderate, and vigorous) for each subject, and included these averages as predictors in the initial models of MVPA. To avoid correcting for the outcome, these averages were calculated only for days without valid sport. All but 9 individuals had at least one such day. It is impossible to calculate a baseline activity level for a subject who has sport every day, so they were excluded from analyses that used baseline activity as a predictor.

*Statistical Methods*

All statistical analyses were conducted using Statistical Analysis Software (SAS, Cary, NC, USA) version 9.3. Participant characteristics were described using means and standard deviations when normally distributed; for skewed variables the 5th and 95th percentile are also provided. Comparisons between subjects (Table 1) used Wilcoxon’s two-tailed rank-sum test; comparisons between sport episodes (Tables 2, 3 and 5) used generalized linear models (PROC GENMOD in SAS.) Nonzero MVPA was modeled as a negative-binomial rate, with total MVPA from the episode as the outcome and the log of length of the episode as offset. This adequately captures the variation that is associated with sport periods of different lengths: the amount of MVPA per minute of a 300-minute episode can be estimated more precisely than that of a 10-minute episode. Zero MVPA, i.e. no time spent in MVPA throughout the entire sporting period, was modeled using logistic regression on all episodes. Because of the greater variability in short episodes, they may have an increased rate of zeroes even though they tend to produce more MVPA per minute (see Table 3). Thus, all multivariable models of zero MVPA were corrected for episode length regardless of statistical significance.

Because of the possibility of effect heterogeneity, initial models were stratified by gender of the subject and team status of the sporting episode. (Data not shown.) The final model for all subjects and episodes (Table 3) included interaction terms for those predictors that had been significant for one group and not the other. Statistically significant (p<0.10) predictors were included in the initial multivariable model. All models were corrected for parental education, study center, subject age, and gender; other predictors were removed in order of least significance until all that remained were significant at the 0.05 level. Retention of categorical predictors was done on the basis of type 3 test for null hypothesis (probability that all categories were equal) and all levels of a predictor were either kept or dropped together.

Freedson, P. P., D; Janz, KF (2005). "Calibration of accelerometer output for children." Med Sci Sports Exerc **37**(11): 523-530 (Suppl).

Kromeyer-Hauschild, K. W., M; Kunze, D; Geller, F; Geiß, HC; Hesse, V; von Hippel, A; Jaeger, U; Johnsen, D; Korte, W; Menner, K; Müller, G; Müller, JM; Niemann-Pilatus, A; Remer, T; Schaefer, F; Wittchen, HU; Zabransky, S; Zellner, K; Ziegler, A; Hebebrand, J (2001). "Body-mass-Index für das Kinder- und Jugendalter unter Heranziehung verschiedener deutscher Stichproben. (Percentiles of body mass index in children and adolescents evaluated from different regional German studies.) In German; abstract in English; tables legible without German." Monatsschr Kinderheilkd **149**(8): 807-818.

Pfitzner, R. G., L; Heinrich, J; von Berg, A; Klümper, C; Bauer, CP; Koletzko, S; Berdel, D; Horsch, A; Schulz, H (2013). "Physical Activity in German Adolescents Measured by Accelerometry and Activity Diary: Introducing a Comprehensive Approach for Data Management and Preliminary Results." PLOS One **8**(6): e65192.

Smith, M. S., H (2014). Contribution of School Physical Education to Total Activity Volume in German Adolescents. Handbook of Physical Education Research: Role of School Programs, Children's Attitudes and Health Implications. R. Todaro. New York, Nova**:** 203.
